# Comparative Expression of *RAGE* and* SOX2* in Benign and Malignant Prostatic Lesions

**DOI:** 10.31557/APJCP.2019.20.2.615

**Published:** 2019

**Authors:** Tarek Aboushousha, Rana Lashen, Khadega Abdelnaser, Noha Helal, Mona Moussa, Zeinab Omran, Samir Eldahshan, Hossam El Ganzoury

**Affiliations:** 1 *Department of Pathology, *; 3 *Department of Urorology Theodor Bilharz Research Institute, Cairo,*; 2 *Faculty of Biotechnology, University of Modern Sciences and Arts, Giza, Egypt. *

**Keywords:** RAGE, SOX2, prostate, cancer, immunohistochemistry

## Abstract

**Background:**

Prostate cancer (PCa) is a common health problem in elderly. RAGE (Receptor for advanced glycation end products) is overexpressed in multiple human cancers. *SOX2* (Sex-determining region Y box 2) also functions as an oncoprotein and promotes cancer progression but the mechanisms involved remain largely unknown.

**Aim:**

The current study investigated the expression patterns of *RAGE* and *SOX2* in benign and malignant prostate samples in correlation with the histopathological findings in order to evaluate their role as prognostic markers or therapeutic targets.

**Methods:**

Immunohistochemical staining for *RAGE* and *SOX2* antibodies was applied on 87 prostatic biopsies [16 of prostatitis, 20 of benign prostatic hyperplasia (BPH) and 51 of PCa].

**Results:**

Expression of *RAGE* and *SOX2* (percentage of positive cells) was significantly higher in PCa lesions compared with prostatitis (p<0.01) and BPH (p<0.0001) and was also significantly higher in prostatitis compared with BPH lesions (p<0.01). Also, percentage of positive *RAGE* and *SOX2* cells showed a significant stepwise increase from Gleason Grade 3 to Grade 5 and were significantly higher in high Gleason Scores (≥8) compared to lower Scores (≤7) with statistical significance (p=0.001).

**Conclusion:**

RAGE and *SOX2* were up-regulated in prostate cancer lesions, mainly in advanced grades, suggesting an active role of both antigens in the development and progression of prostate cancer and expecting the possibility of their use as therapeutic targets.

## Introduction

Prostate cancer (PCa) is a common health problem that in the majority of cases starts to develop at the age of 50 years, reaching its peak at 60–70 years of age (Boyle et al., 2003). While highly curable if localized, patients with metastatic disease have a 5-year survival rate of only 31% (Takahashi et al., 2007). As no reliable means of identifying patients at risk of metastatic dissemination are currently available (Briganti et al., 2014). A better understanding of the mechanisms underlying the spread of prostate cancer cells should aid the development of treatment strategies that improve outcomes for patients with advanced disease (Bae et al., 2016).

RAGE (Receptor for advanced glycation end products) is a cell surface molecule and a member immunoglobulin superfamily (Alexiou et al., 2010). RAGE has multiple ligands; its major one is advanced glycation end products (AGEs) and AGE-RAGE interaction is implicated in development, homeostasis, inflammation, neurodegenerative disorders, tumors, and pathogenesis of various diseases (Yan et al., 2009). It is likely that abnormal stimulation of AGE-RAGE affects cell cycle genes controlling the G1/S phase transition, increases the number of cells in the S phase of the cell cycle (DNA synthesis phase) and decreases the percentage of cells in G1 phase (cell growth phase) (Kim et al., 2008), so this interaction can be involved in the development, growth and metastasis of a number of tumor types including prostate cancer (Sims et al., 2010).

The embryonic stemness gene *SOX2* [Sex-determining region Y box 2] is one of the key members of the Sox family that encodes a series of transcription factors. *SOX2* functions as an oncoprotein and promotes cancer progression and focused on its positive contribution to many physiological processes of cancer cells, such as proliferation and growth, cellular migration and invasion, maintenance of stemness, anti-apoptotic activity, chemoresistance, metastasis and tumorigenesis, however, the mechanisms involved remain largely unknown, both in prostate and in other cancers (Weina and Utikal, 2014).

In this study, we analyzed immunohistochemical expression of *SOX2* and *RAGE* expression in benign and malignant prostatic tissue samples and investigated their correlation with Gleason Grade, Gleason Score and Grade Group of prostate cancer cases.

## Materials and Methods

We collected archival prostatic specimens and clinical pathological data related to 87 patients who had chronic urologic complaint and came to seek medical advice at Urology Department, Theodor Bilharz Research Institute, in the interval from January 2016 to December 2017, where clinical examination and radiological investigations were done, then cystoscopic or transrectal ultrasound-guided biopsies were taken. 

Routine histopathological processing and diagnosis were made at Pathology Department, Theodor Bilharz Research Institute, using hematoxylin and eosin stain. Pathology reporting sheet includes diagnosis (benign: hyperplasia/ prostatitis or malignant) and scoring of malignant cases according to Gleason’s criteria. Then immunohistochemical studies were applied on archival prostatic tissue using *RAGE* and *SOX2* antibodies.

Prostate cancer patients under study did not receive hormone therapy, che¬motherapy or radiotherapy. Serum PSA (Serum prostate-specific antigen) levels were measured for all studied cases.

This study was approved by our institutional ethics committee.

Study groups were categorized as: Prostatitis: 16 cases; Benign prostatic hyperplasia (BPH): 20 cases; and Prostate cancer (PCa): 51 cases.

Prostate cancer samples were further classified according to Gleason Classifications (Moch et al, 2016):

• Gleason Grade (Grades 1-5): According to degree of tumor differentiation.

• Gleason Score (Scores 2-10): Determined by adding up the two most prevalent Gleason Grade numbers.

• Gleason Grade Group (Groups 1-5): A newly categorized grading system that is based on Gleason score.

*Immunohistochemical technique*

Tissue sections were processed into 3 μm thick sections. Unmasking of the antigen was performed with 10 ml sodium citrate buffer, pH 6.0, at 90^o^C for 30 minutes. Sections were incubated in 0.03% hydrogen peroxide for 10 minutes at room temperature to remove endogenous peroxidase activity and then in blocking serum (0.04% bovine serum albumin, A2153, Sigma-Aldrich, Shanghai, China, and 0.5% normal goat serum X0907, Dako Corporation, Carpinteria, CA, USA, in PBS) for 30 minutes at room temperature. Sections were incubated overnight at 4°C in humid chamber with the primary antibodies: Anti-RAGE antibody (A11) (Santa Cruz Biotechnology, Catalog no. sc- 80652, CA, USA) and Anti- *SOX2* antibody (Abcam, Catalog no. ab97959, Cambridge, MA, USA) at an optimal dilution of 1:100. Sections were then washed three times for 5 min in PBS. Non-specific staining was blocked 5% normal serum for 30 min at room temperature. Finally, staining was developed with diaminobenzidine substrate and sections were counterstained with hematoxylin. PBS replaced *RAGE* and *SOX2* antibody in negative controls. 

**Table 1.** Differences in Mean Age, Mean PSA Level, RAGE Expression and SOX2 Expression among Studied Groups

Diagnosis	Age Mean±S.D.	PSA Mean±S.D.	Percentage of positive cells Mean±S.D.	
			RAGE expression	SOX2 expression
Prostatitis (16)	65.33±4.50	20.53±9.86	33.91±6.17	50.00±16.73
BPH (20)	68.30±4.05*	10.90±2.03**	18.57±4.35**	37.00±10.59**
PCa (51)	68.97±5.33*	72.88±20.39**##	88.33±1.43##**	70.56±17.56##**

*, Significant difference compared to prostatitis (p=0.05); **, High significant difference compared to prostatitis (p=0.01); ##, High significant difference compared to BPH (p=0.0001).

**Table 2:** Relation of Gleasons' Scores to *RAGE* and *SOX2* Expression

Item	Percentage of positive cells Mean±S.D.	
	RAGE expression	SOX2 expression
Gleason Score		
Grade 3 (32)	84.37±9.65	64.28±17.62
Grade 4 (12)	95.00±4.26^	80.00±11.18^
Grade 5 (7)	100.00±0.00 ^@^	100.00±0.00 ^@^
Gleason Total Score		
Score 6 (25)	82.85±9.37	64.13±17.16
Score 7 (7)	92.50±2.76*	67.85±18.67
Score 8 (13)	100.00±0.00**	79.09±10.44**
Score 9 (3)	97.50±2.67**	95.00±0.00**
Score 10 (3)	100.00±0.00**	100.00±0.00**
Gleason Grade Group		
Grade Group 1 (25)	82.85±9.37	64.13±17.16
Grade Group 2 (7)	92.50±7.76^#^	58.33±27.53
Grade Group 3 (12)	93.12±3.88^##^	75.01±5.77^#^
Grade Group 4 (2)	100.00±0.00 ^@^	83.11±10.15
Grade Group 5 (5)	97.50±2.67^##^	95.00±0.0 ^@^
P value ANOVA	0.0001	0.005

^, High significant difference with score 3 (p<0.01); *, Significant difference with total score 6 (p<0.05); **, High significant difference with total score 6 (p<0.001); #, Significant difference with Grade Group 1 (p<0.05); ##, High significant difference with Grade Group 1 (p<0.001); @, t-test could not be performed (as S.D. = Zero).

**Table 3:** Correlations between Expression of *RAGE* and *SOX2 *with Different Studied Parameters of PCa

Spearman's rho		PSA	Gleason Grade	Gleason Sore	Gleason Grade Group
*RAGE *% of positive cells	Correlation Coefficient	0.559**	0.528**	0.759**	0.492**
	N	87	51	51	51
*SOX2* % of positive cells	Correlation Coefficient	0.406**	0.504**	0.521**	0.529**
	N	87	51	51	51

*, Correlation is significant at the 0.05 level (two-tailed); **, Correlation is significant at the 0.01 level (two-tailed); %, percentage; N, number of cases.

**Figure 1:**
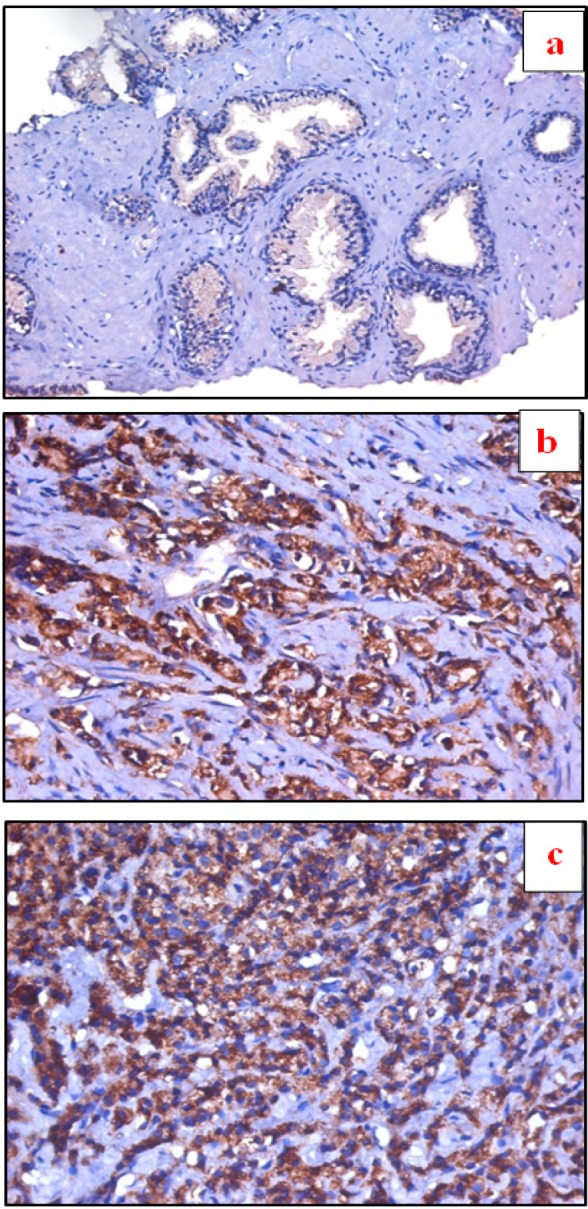
a, Section in Benign prostatic hyperplasia, showing negative RAGE expression in prostatic glands (IHC for *RAGE*, DAB X100); b, Section in Prostatic adenocarcinoma, Gleason Grade 3, showing moderate membrano-cytoplasmic RAGE expression in prostatic glands (IHC for *RAGE*, DAB X100); c, Section in Prostatic adenocarcinoma, Gleason Grade 5, with marked membrano-cytoplasmic positive expression for RAGE (IHC for *RAGE*, DAB X100)

**Figure 2.**
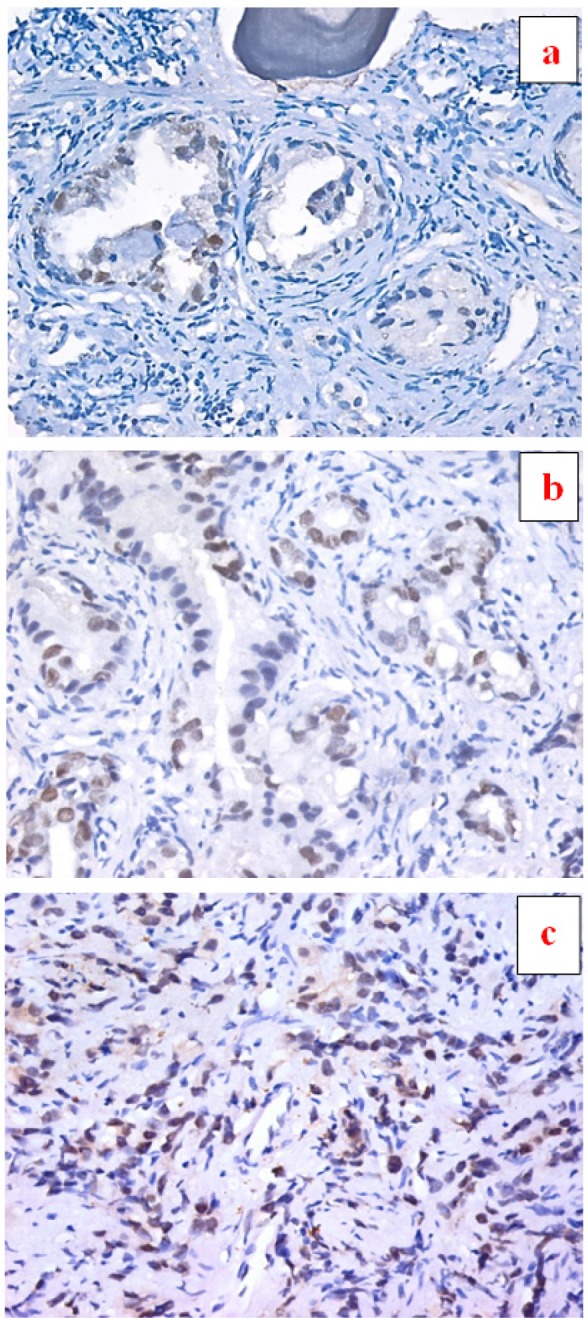
a, Section in Benign prostatic hyperplasia, showing prostatic acini lined by a double layer of epithelial cells with discrete positive nuclear expression for *SOX2* in epithelial cells (IHC for *SOX2*, DAB X 200); b, Section in Prostatic adenocarcinoma, Gleason Grade 3, showing scattered malignant prostatic acini lined by a single layer of epithelial cells with moderate positive nuclear expression for *SOX2* in epithelial cells (IHC for *SOX2*, DAB X 200); c, Section in Prostatic adenocarcinoma, Gleason Grade 5, showing infiltrating single files of epithelial cells with moderate nuclear expression for *SOX2* (IHC for *SOX2*, DAB X100)

*Interpretation of immunostaining*

The expression of *RAGE* and *SOX2* was semi-quantitatively estimated as brown membrano-cytoplasmic immunostaining (for *RAGE*) and brown nuclear immunostaining (for *SOX2*), which was calculated as the proportion of positive cells to the total number of cells calculated in 10 successive high power fields (percentage of positive cells). 

The sections were examined by using light microscope [Scope A1, Axio, Zeiss, Germay]. Photomicrographs were taken using a microscope-camera [AxioCam, MRc5, Zeiss, Germany]. 

*Statistical analysis*

SPSS for Windows, version 20, was used for statistical analysis (IBM Corporation, Armonk, NY, USA). Results were given as mean ±SD. Distribution of negative and positive cases was studied with cross tables (Pearson’s Chi square-test). Spearman rank correlation coefficient was used to investigate a possible correlation of *RAGE* or *SOX2* expression with tumor grade. The comparisons of quantitative variables were performed between two groups using ANOVA and student t-tests. A p value of < 0.05 was considered of statistical significance.

## Results

In our study, the mean ages of PCa and BPH were 68.30 and 68.97 years respectively which showed significant difference with the mean age of prostatitis cases (65.33years) with p=0.05. Mean of serum PSA level was higher in PCa lesions (72.88 mg/ml) compared with prostatitis (20.55 mg/dl ) and PBH (10.90 mg/dl) with significant differences (p<0.01 and <0.0001 respectively) (Table 1).

Expression of *RAGE* and *SOX2* (percentage of positive cells) was significantly higher in PCa lesions compared with prostatitis (p<0.01) and BPH (p<0.0001) and also was significantly higher in prostatitis compared with BPH lesions (p<0.01) (Table 1) (Figure 1 and 2)

By comparing the different grades of PCa according to Gleason’s classification; percentage of positive RAGE and *SOX2* cells showed a significant stepwise increase from Gleason Grade 3 to Grade 5. Also, by comparing Gleason Score of PCa; expression of RAGE and *SOX2* showed significantly increase in high Gleason Scores (≥8) compared to lower Scores (≤7) with statistical significance (p=0.001). Furthermore, by comparing the different Gleason Grade Groups of PCa according to the new Gleason classification; expression of RAGE and *SOX2* was significantly increased in high Gleason Grade Groups (≥3) compared to lower Groups (≤2) with statistical significance; being tested by ANOVA (Table 2).

Our study showed a significant positive correlation between RAGE and *SOX2* percentage of positive cells with different examined Gleason’s classifications (Gleason Grade, Score and Grade Group), but no significant correlation with PSA level (Table 3).

## Discussion

In Egypt, according to Registry Program 2008-2011, prostate cancer is the sixth cancer among male malignancies, with an incidence of 4.27% (Ibrahim et al., 2014). 

Serum prostate-specific antigen (PSA) is a protein produced by normal and malignant prostate gland and is a widely used parameter for early detection and monitoring of prostate cancer. Consistent with the fact that in addition to prostate cancer, prostatitis and BPH can cause elevation in man’s PSA level; we found an increase in mean PSA value in prostate cancer compared with prostatitis and BPH. Naz et al., (2004) observed that a majority of prostate cancer cases had serum PSA >20 ng/ml and 50% of BPH cases had serum PSA in the gray zone (4.1-20 ng/ml). Meanwhile, PSA levels did not show significant correlations with RAGE or *SOX2* expression in our studied prostatic lesions.

The AGE-RAGE interaction plays a crucial part in the development of prostate cancer. Ishiguro et al., (2005) stated that inhibiting this interaction has potential as a new molecular target for cancer prevention or therapy. Several reports had revealed an association between the expression of RAGE and development of cancers, as Aboushousha et al., (2016) found that RAGE expression was up-regulated in human gastric adenocarcinoma and Kuniyasu et al., (2003) described that RAGE production was enhanced in metastatic compared to non-metastatic prostate cancer and healthy prostate tissue.

In our study, we found significant increase in mean percentage of RAGE positive cells in prostate cancer tissue compared with prostatitis and BPH, which matches results of Lu et al., (2010) who reported a significant higher expression of RAGE in the prostate cancer than in normal prostate tissue by immunohistochemistry, western blot analysis and real-time quantitative PCR. Ishiguro et al., (2005) found that RAGE mRNA expression was up-regulated in prostate cancer tissues and also found that untreated primary prostate cancer tissue and hormone-refractory prostate cancer tissue showed significantly higher RAGE mRNA expression than normal prostate tissues. In a study performed by Aboushousha et al., (2018) they reported a significant higher RAGE expression in hepatic dysplasia and hepatocellular carcinoma lesions than in non-tumor hepatic tissue. Similarly, Amornsupak et al., (2017) found stronger RAGE immunoreactivity in the breast cancer tissue sections than in that in the benign disease tissue. Collectively, these findings suggest a role played by RAGE in development and growth of cancer lesions.

According to our results, RAGE expression showed a positive significant correlation with each of Gleason Grade, Gleason Score and Gleason Grade Group. We found a significant increase in mean percentage of RAGE positive cells in higher Gleason Grades (≥4) compared with lower Grade 3, and in higher Gleason Scores (≥ 8) compared with lower Scores (≤7) and also in higher Gleason Grade Groups ((≥3) compared with lower Groups (≤2). RAGE overexpression had been reported in advanced grades and stages in diverse types of malignant tumors. Aboushousha et al., (2016) found a significant correlation between RAGE expression with advanced stages and lymph node metastasis of gastric adenocarcinoma. Nankali et al., (2016) reported higher *RAGE *expression in high-stage and aggressive breast cancer tumors. Sasahira et al., (2005) demonstrated higher *RAGE* expression in colorectal adenomas with severe atypia. In contrast; Aboushousha et al., (2018) detected higher expression of RAGE in low grades of hepatocellular carcinoma compared with high grades, moreover, Ishiguro et al., (2005) did not find a significant difference in *RAGE* mRNA expression according to both grade and stage in their prostate cancer samples.

*SOX2*, an important transcription factor, has been shown to be over-expressed in most human cancer types, including prostate cancer. It plays a key role in cell self-renewal, differentiation, proliferation and apoptosis in stem cells and in cancer cells (Lin et al., 2012).

Bae et al., (2010) and Jia et al., (2011) reported an increase in percentage of *SOX2* positive cells in prostate neoplastic tissue compared with BPH, which matches our results. In our study, we demonstrated a significant higher percentage of *SOX2* positive cells in prostate cancer compared with prostatitis and BPH lesions; however, Ugolkov et al., (2011) reported an increase in expression of *SOX2* in both benign and malignant prostate tissue, but only in a small percentage of cells (10%). So it is possible that *SOX2* participates in the development and regulation of both hyperplastic and malignant prostatic tissues. Meanwhile Russo et al., (2016) demonstrated distinct *SOX2* expression in the basal cell layer of normal prostate glands and its absence in most of the neoplastic epithelia, with the exception of a few foci of low-grade and high-grade prostate cancer, which agrees with the notion that prostates lose basal cells during cancer progression. Taken together, these controversial findings have indicated that the dynamics of *SOX2* expression in prostate cancer are largely unknown and there is a need for further studies to elucidate its role in prostate cancer progress.

In accordance with previous studies (Bae et al., 2010; Jia et al., 2011; Kregel et al., 2013) which reported a correlation between *SOX2* expression and both of Gleason Grade and Gleason Score, we found a statistical significant increase in percentage of *SOX2* positive cells in higher Gleason Grades, Gleason Scores and Gleason Grade Groups compared with lower ones. Since a higher Gleason grade indicates a worse prognosis, so it is suggested that *SOX2* may contribute to the tumorigenesis of prostate cancer and may play an important role in the clinical progress of prostate cancer. Bae et al., (2016) postulated that, the expression of *SOX2* in prostate tumors has been thought to promote a less differentiated embryonic stem cell tumor phenotype; that confers a worse disease prognosis. 

In the present study, we found a positive significant correlation between percentage of *SOX2* positive cells and each of Gleason Grade, Gleason Score and Gleason Grade Group. Many previous studies reported an association between *SOX2* expression and advanced stages in several human tumors. Yang et al., (2013) reported that *SOX2* expression was associated with clinical stage and lymph node status in patients with small cell lung cancer. Tang et al., (2013) suggested that *SOX2* expression was significantly associated with clinical stage, lymph node metastasis and recurrence in laryngeal squamous cell carcinoma. Zhang et al., (2010) reported that patients with strong *SOX2* expression showed deeper invasion and advanced clinical stages compared to patients with low *SOX2* expression in gastric cancer. Huang et al., (2014) reported a close association between *SOX2* expression with clinicopathological parameters in breast cancer; including high histological grade, large tumor size, molecular subtypes, negative hormone receptor status and high proliferation index. 

 In conclusion, *RAGE* and *SOX2* were up-regulated in prostate cancer; mainly in cancers with a worse prognosis which have higher Gleason Grade, higher Gleason Score and higher Gleason Grade Group. So, we suggest a role played by *RAGE* and *SOX2* in the progression of prostate cancer that they can be used as predictive tools or therapeutic targets in prostate cancer.
